# The coloniality of age: Navigating the chronopolitics of Black childhood

**DOI:** 10.1111/1468-4446.13141

**Published:** 2024-08-22

**Authors:** Callum Stewart

**Affiliations:** ^1^ The University of Melbourne Parkville Victoria Australia; ^2^ The University of Cambridge Cambridge UK

**Keywords:** coloniality of time, counter‐narrative, decolonisation, otherwise worlds, racialised time, temporal agency

## Abstract

For Black, Indigenous, and other colonised peoples, decolonisation and racial justice are urgent imperatives, but their demands are often dismissed as utopian, impossible, or otherwise out‐of‐time. This article therefore introduces the coloniality of age as a theoretical framework that aims to open up possibilities for otherwise worlds. Departing from established accounts of the coloniality of time, the coloniality of age grounds the analysis of racialised time in the chronopolitical formations of *tempus nullius* and the paternalistic paradigm. Alongside the doctrine of *terra nullius* or ‘uninhabited land’, the doctrine of *tempus nullius* or ‘uninhabited time’ works to deny Black peoples the ability to make and remake history on their own terms. Supplementing theories of the barbarian other, the paternalistic paradigm identifies patriarchal father/child relations as a conceptual and historical precedent to race. The coloniality of age directs the analysis to the temporal limits of coloniality. I argue that the temporal limits of coloniality are constituted by Black childhood; the coloniality of age figures Black childhood as an age with no future. This framework is then applied to analyse young Black peoples' counter‐narratives of Black childhood. The counter‐narratives of being ‘stuck’, ‘growing up’, the ‘pace’ of racism, and ‘regressing’ centre the temporal agency of Black children as they navigate the chronopolitics of Black childhood. Each of these counter‐narratives unsettles the coloniality of age. Read together, the counter‐narratives tell a larger story of Black children confronting the temporal limits of coloniality, refusing the terms of White futurity, and instead opting to grow otherwise. The article concludes that Black childhood might be reframed as an age with otherwise futures beyond the temporal limits of coloniality.

## 1 INTRODUCTION

Decolonisation and racial justice often appear impossible, utopian demands in a world built on the theft of the lives, lands, and futures of Black, Indigenous, and other colonised peoples. This article therefore asks, what are the temporal limits of coloniality? Throughout White Britain and its empire, debates over decolonisation and racial justice predominantly take coloniality as their self‐evident frame of reference. This frame of reference immediately locates decolonisation and racial justice out‐of‐time (Mills, 2014; Rifkin, 2017). By directing the analysis to the temporal limits of coloniality, this article aims to open up submerged possibilities for the repatriation of Indigenous life and land, the abolition of racialised systems of oppression, and the construction of otherwise worlds (Crawley, 2020; Tuck & Yang, 2012). Towards this aim, this article introduces the coloniality of age. The coloniality of age is a theoretical framework that locates racialised time within the colonial chronopolitical formations of age from which it emerges. This article deploys the coloniality of age to analyse young Black peoples' counter‐narratives of their childhoods. It explores the temporal limits of coloniality by asking: How do young Black people navigate these limits? The futurity of Black childhood is often figured as a central concern in debates over decolonisation and racial justice in Britain; in debates over police violence, incarceration rates and youth crime, school exclusion rates and ‘decolonising’ the curriculum, welfare support and poverty, and immigration and deportations, the lives and futures of Black children are at stake. In each of these debates, representations of Black children's futurity or lack thereof shape the terrain of British politics. This analysis of the chronopolitics of Black childhood works to undo the coloniality of age and gestures towards hidden possibilities for growing otherwise.

This article departs from accounts of the coloniality of time (Mignolo, 2012; Quijano, 1993, 2000). The coloniality of time describes the colonial construction of ‘time’ as an abstract and universal history, but risks reifying the colonial matrix of power (Beard, 2016; Rifkin, 2017). Here, I foreground age to theorise the complex relational and embodied nature of racialised time in new ways, holding time open to the self‐determination of Black, Indigenous, and other colonised peoples. The coloniality of age introduces, brings together, and extends two insightful but often overlooked research threads in the analysis of racialised time. First, age situates this article in relation to Al‐Saji (2013) and Ngo's (2021) Fanonian phenomenological analyses of White and Black peoples' respective lived experiences of the past, present, and future. I supplement spatial analyses of the doctrine of *terra nullius* by developing Ngo's (2021, p. 201) suggestion that coloniality is also structured through the doctrine of *tempus nullius*, or ‘uninhabited time’. I argue that *tempus nullius* forms the conditions of possibility for the coloniality of age. Second, age situates this article in relation to Nakata's (2015) analysis of the paternalistic paradigm, which establishes the patriarchal adult/child relationship as the basis for conceptualising race. I supplement spatial analyses of the barbarian other by presenting the paternalistic paradigm as an historical precedent to the coloniality of age (Nandy, 1983; Rollo, 2018a, 2018b). I argue that the paternalistic paradigm structures the coloniality of age's trajectories of growth upwards from a Black past towards White futures.

The coloniality of age allows this article to interrogate the temporal limits of coloniality through the chronopolitics of Black childhood. Chronopolitics refers to the multiple networks of symbolic and material power relations through which conflict over divergent histories, futurities, processes of becoming and possibilities for action take place (Mills, 2020; Rifkin, 2017). Rather than trying to make Black children intelligible in ways that reify coloniality, the chronopolitics of Black childhood examines constructions of Black childhood in order to better understand the temporal politics of racial justice and decolonisation (Burman, 2019). Building on analyses of colonial constructions of Black childhood (Breslow, 2019; Nakata & Bray, 2023; Rollo, 2018b; Shange, 2019), I argue that Black childhood constitutes the temporal limit of coloniality; the coloniality of age figures Black childhood as having no future.

This article analyses the counter‐narratives of young Black people to understand the chronopolitics of Black childhood from a fresh perspective. As Kromidas (2019a, p. 21) argues, ‘abstract theorisations and deconstructions may counter dominant narratives but they do not invite new modes of being and relating’. Building on recent work across childhood studies, Black studies, Indigenous studies, and queer theory (Conrad, 2020; English, 2013, 2018; Kromidas, 2019a, 2021, 2022; Pacini‐Ketchabaw & Kummen, 2016; Rifkin, 2017; Shange, 2019), these counter‐narratives show how Black children experience and critically navigate the temporal limits of coloniality in their daily lives. The young Black people narrated these experiences in terms of being ‘stuck’, ‘growing up’, of being unable to ‘outpace racism’, and of ‘regressing’. Separately, the counter‐narratives reflect the unique experience and temporal agency of each young Black person. Together, the counter‐narratives tell a larger story of Black children coming face‐to‐face with the temporal limits of coloniality, and refusing the terms of White futurity. Reflecting on the chronopolitics of Black childhood through these counter‐narratives allows us to see how the coloniality of age shapes and limits possibilities for the future, while also holding processes of becoming open to other pathways.

## 2 THE COLONIALITY OF TIME

Decolonial theory conceives of race as the fundamental principle through which the colonial matrix of power is organised (Quijano, 1993, 2000). The colonial matrix of power is an overarching heterarchical and global structure produced through colonial histories that continues to shape global relations well after processes of formal decolonisation in large parts of Africa, Asia, and Latin America (Maldonado‐Torres, 2007, p. 243). Decolonial theory defines race as the codification and naturalisation of the colonial difference between the coloniser, produced as a human subject racialised as White, and the colonised, produced as a subhuman object and racialised at its lowest as Black (Wynter, 2003). This definition of race allows decolonial theory to deconstruct the coloniality of time. The coloniality of time describes time as a racialised universal, linear, progressive and teleological history, abstracted from body and nature (Mignolo, 2012; Quijano, 1993, 2000).

The coloniality of time naturalises a racialised distinction between body and nature, identified with colonised peoples, and the rational subject, identified with colonisers. Coloniality produces time as a universal abstract separated from the movements of nature (Mignolo, 2012). The colonisation of the Americas from 1492 transformed humanity's relation to nature within the European colonial imaginary, epitomised within the Cartesian construction of the rational subject. The rational subject abstracts the mind from body and nature, concealing the sociopolitical and geopolitical place from which knowledge is produced (Maldonado‐Torres, 2007; Mignolo, 2011). The rational subject is therefore conceived as capable of viewing the natural world from the ‘zero point’; the perspective of God as a detached, neutral observer (Castro‐Gómez, 2021; Mignolo, 2011). ‘Time’ is consequently produced as an abstract and universal category written and recorded in human‐produced calendars and clocks, rather than being measured through the embodied experience of nature. The movements of nature are objectified in this anthropocentric worldview; body and nature are no longer conceived as living‐in‐place but as fixed and universal objects of colonial knowledge (Mignolo, 2012, p. 20).

The coloniality of time further naturalises a racialised distinction between the primitive past, inhabited by colonised peoples, and the civilised present and future, inhabited by colonisers (Mignolo, 2012; Quijano, 1993, 2000). Coloniality produces human history as a universal, linear, progressive, and teleological history from primitive to civilisation. According to Quijano (Quijano, 1993, p. 143, 2000, pp. 546–547), the colonisation of the Americas also transformed humanity's conception of history, which came to fruition in the Enlightenment. Mignolo (2012) unpacks this emergent conception through Kant's seminal *Idea of Universal History from a Cosmopolian Point of View*. Kant reconstructed the abstract universal of time into a secular history of the rational subject, in which ‘all natural capacities are destined to *evolve* completely to their natural end’ (Kant 1784 cited in Mignolo, 2012, p. 79, italics by Mignolo). The rational subject is therefore conceived as capable of viewing the world from a temporal zero‐point, as if looking back on nature from the end of history (Mignolo, 2012; Quijano, 2007). History is produced as a fixed and static object of colonial knowledge in which past, present, and future are separable by reference to progress.

Genealogies of abstract ‘time’ may deconstruct the dominant temporal narratives of coloniality, but they also foreclose external reference points and elide other possibilities for action. Quijano (1993, 2000) and Mignolo (2011, 2012) aim to establish colonisers and colonised peoples as coeval by locating modernity's history in the universal context of coloniality. However, Quijano and Mignolo consequently risk presenting the colonial matrix of power as a totalising global structure; it often appears that coloniality determines colonisers' and colonised peoples' possibilities for action. The construction of modernity/coloniality through a singular universal history precludes possibilities that emerge from other temporal formations, or subsumes them into an all‐encompassing colonial structure (Rifkin, 2017, pp. 10–11). This risks reifying the colonial matrix of power in ways that deny more transformative possibilities for racial justice and decolonisation that already exist in the lifeworlds of Black, Indigenous, and other colonised peoples' (Beard, 2016; Martineau & Ritskes, 2014; Tuck & Yang, 2012). The imposition of the coloniality of time is not complete nor universal; Indigenous, Black, and other colonised communities have always had their own distinct histories and futures beyond the colonial matrix of power (English, 2013, 2018). In the following two sections, I provide the theoretical framework for the coloniality of age, made up of the doctrine of *tempus nullius*, and the paternalistic paradigm. This framework locates ‘time’ within intersecting, dynamic and complex networks of chronopolitical relations.

## 3 THE DOCTRINE OF *TEMPUS NULLIUS*

In his analysis of the White temporal imaginary, Mills (2014, p. 31) argues that ‘The White settler state “sets the historical chronometer” at zero to signal that before its arrival, no history has taken place’. Colonisation is here constructed as the beginning of human history. This rupture racialises what Conrad (2020, p. 12) terms ‘temporal agency’, the ability to use time appropriately, to make history with time. White colonisers are produced as the ‘masters of their own time’, able to bend time to their will. Black and Indigenous peoples are, in contrast, produced as a ‘dying race’ naturally ‘mastered by time’ (Mills, 2014, p. 31). Ngo (2021, p. 201) therefore describes colonialism as founded not only ‘on the basis of *terra nullius*, but also *tempus nullius*—uninhabited time, time not utilised or made use of, time that therefore does not register as such’. I unpack the deep theoretical potential of this concept here, arguing that *tempus nullius* forms the conditions for the coloniality of age.

Al‐Saji (2013) argues that Fanon's racialisation as Black structures his temporal experience of the past. Prior to his racialisation, Fanon is able to find meaning in history and orient himself towards the future through his own corporeal schema. Following his racialisation, however, ‘the corporeal schema crumbled, its place taken by a racial epidermal schema’ (Fanon, 2008, p. 84). With regards to age, a racial schema of *tempus nullius* relocates and traps Fanon into a Black past. Produced by the White man, the Black past is ‘a past of stereotyped remnants, isolated fragments and colonised distortions extrapolated back from their [colonised peoples’] oppressed and alienated state under colonialism’ (Al‐Saji, 2013, p. 6). The Black past is imposed on Fanon through colonial structures; Fanon finds himself ‘battered down by tom‐toms, cannibalism, intellectual deficiency, fetishism, racial defects, slave‐ships, and above all else, above all: “Sho’ good eatin’” (Fanon, 2008, pp. 84–85). Upon his interpellation as Black, Fanon no longer experiences the past as past, but as ‘a fixed and overdetermining dimension of the present’ (Al‐Saji, 2013, p. 5). The Black past is not a past that can be reanimated to reimagine the present and future. It is a ‘closed past incapable of development on its own terms and cut off from the creativity that gives rise to an open future’ (Al‐Saji, 2013, pp. 6–7). Colonial violence instead works to orient Black bodies towards the past. The question, ‘where do you come from?’, for example, stops Black bodies from moving through the world. It instigates a search for suspicious origins and may habitually orient Black bodies towards the past (Ahmed, 2006, p. 142; Ngo, 2021, pp. 196–197). The Black past is inhabited by Fanon. *Tempus nullius* becomes Fanon's ontological reality, rather than a series of misrepresentations of a distant past.

In a similar manner, Ngo (2021) argues that subjects' racialisation as White structures their temporal experience of the future. Ngo explores White subjects' temporal experience through Mills' (2007) theory of White ignorance. White ignorance is grounded in a refusal to acknowledge colonial and racist histories, instead reproducing coloniality and racism through the ‘management of memory’ enabled by *tempus nullius* (Mills, 2007, p. 28). Ngo (2021, p. 199) argues that White ignorance entails an important temporal dimension; it is produced through ‘a leaving behind and a moving on’. White ignorance may be produced through a racialised forgetting and narrative omission (Mills, 2007). White subjects may deflect accusations of racism by pleading ignorance of the colonial and racist histories which give their habits meaning. Ongoing colonial violence may instead be recast as a singular innocent and natural reaction to the present realities of race, disconnected from the Black past. White subjects produce a temporal distance between themselves and the Black past through the doctrine of *tempus nullius*, disavowing that past as not of their making (Ngo, 2021).

White ignorance may also be produced through a racialised remembering that constitutes the narrative itself. Some White Australians, for example, are wedded to the celebration of Australia Day on January 26 in commemoration of the beginning of the British ‘settlement’ of Australia in 1788. While this may appear as a nostalgic orientation towards the past, it functions to reinstate White settler narratives of colonial conquest and sovereignty. White subjects may reimagine the past as the future ‘since its taking up expresses a re‐animation, a re‐enlivening that brings those pasts forward and casts them anew’ (Ngo, 2021, p. 201). Ngo (2021, p. 201) describes this as a temporal form of ‘ontological expansiveness’ (Sullivan, 2006, p. 10). In its temporal form, White ontological expansiveness allows White subjects to inhabit and move through the past, present, and future as they see fit, while maintaining their ignorance of the coloniality and racism which constitutes those histories (Ngo, 2021, pp. 201–202). White subjects may determine which pasts to bring into the present and future, which to leave behind, and when to move on. Whiteness, then, habitually orients White subjects towards the future. *Tempus nullius* also becomes the ontological reality of White subjects as they make and remake history in ways that sustain coloniality.

The doctrine of *tempus nullius* provides the conditions on which the coloniality of age is built. Drawing on Quijano (2000), Al‐Saji (2013, pp. 6–7) highlights that ‘the elision and repression of precolonial pasts, construed as empty, prehistorical, or primitive lands’ is material, not purely epistemological or psychological. *Tempus nullius* works to deny Black, Indigenous, and other colonised peoples the ability to make and remake their historical relations to the past, present, and future on their own terms. In his embodied experience of Blackness, Fanon finds himself ‘Too late. Everything is anticipated, thought out, demonstrated, made the most of’ (Fanon, 2008, p. 91; cited in Al‐Saji, 2013, p. 7). *Tempus nullius* works to position the White subject as always ahead of the racialised Fanon. The field of possibilities is predetermined by White bodies. Al‐Saji (Al‐Saji, 2013, p. 8) writes,

‘white subjects have already used up these possibilities; they have moved on and left them behind…The field of possibility loses its playfulness and imaginary variability. Though Fanon may sometimes be able to take up the structured possibilities already defined, and follow through their realization according to the routes deposited by the other (to the degree that this is permitted a black body in a white world), he does not see them as allowing variation, as being able to be worked out differently. The structure of possibility allows repetition but not creation or variation; it is a closed map’.

By denying Black, Indigenous, and other colonised peoples' temporal agency, *tempus nullius* limits possibilities for the future to a reproduction of a White future. It clears the way for the paternalistic paradigm to be imposed as the universal model of chronopolitical relations.

## 4 THE PATERNALISTIC PARADIGM

Decolonial theorists typically take the Greco‐Roman geopolitical distinction between the citizen and the barbarian as the historical precedent to race (Maldonado‐Torres, 2007; Mignolo, 2011, 2012; Wynter, 2003). Childhood studies theorists, however, offer ‘the paternalistic paradigm’ as an alternative theoretical framework through which to approach race (Nakata, 2015, p. 15). The paternalistic paradigm establishes the patriarchal adult/child relationship as the basis for conceptualising race. The paternalistic paradigm is based in a naturalised hierarchy between reason and maturity, embodied by the father, and irrationality and immaturity, embodied by the child. It justifies colonial surveillance and interference by claiming White Europeans have a homologous sovereign paternal duty to protect, support, or punish an infantilised colonised race (Nakata, 2015, p. 16). Black and Indigenous peoples have been historically constructed as archetypal ‘child‐races’ (Rollo, 2018a, 2018b). The paternalistic paradigm foregrounds the sociohistorical construction of chronopolitical difference in terms of age and its temporal relations of development or growth ‘upwards’.

Within the paternalistic paradigm, childhood is produced as the constitutive other of the political domain (Nakata, 2015). The paternalistic paradigm defines childhood as a future‐oriented state of becoming and adulthood as a fixed state of being. Within this distinction, the figure of the child is constructed as the future‐adult. Childhood is therefore a site of intense governance and conflict over the future. The governance of childhood stands in for the governance of society (Nakata, 2015; Stewart, 2019). Within this paradigm, childhood development is governed in accordance with ‘developmental time’ or ‘reproductive time’, an adult‐determined universal, linear, progressive, and teleological history from child to adult. Scientific disciplines such as biology and psychology naturalise developmental time. Queer and childhood theorists, however, locate the adult/child relationship within the chronopolitical formations of patriarchy; developmental time is sociohistorically constructed through the assumption of a White, cisheterosexual, middle‐class and abled child as the universal norm (Halberstam, 2005; Kromidas, 2022). Within this paradigm, as Muñoz (2009, p. 95) argues, ‘Racialized kids, queer kids, are not the sovereign princes of futurity’. In order to identify the temporal limits of coloniality, the paternalistic paradigm needs to be further located within the chronopolitical formations of coloniality through which it emerges.

Rollo (2018b) argues that the construction of the child as the constitutive other of the human is integral to the construction of race. For Rollo (2018b, p. 313), ‘the familiar misopedic discourse of childhood inferiority served as a ready‐made scaffold upon which facades of race’ could rationalise the slave trade. Reflecting ‘the conventional view of his day’, American politician William Drayton stated in 1836, ‘the negro is a child in his nature, and the white man is to him as a father’ (Rollo, 2018b, p. 314). The human/child distinction naturalises racial difference; Whiteness and Blackness have been constructed as permanent corporeal markers of a state of mature adulthood and immature childhood respectively (Rollo, 2018b, p. 313). Rollo (2018a, p. 61) further argues that ‘the idea of a telos of progress from animal child to human adult is both a historical and conceptual antecedent of the idea of European civilisation, prefiguring its stories about maturation and progress from cultural ignorance to enlightenment’. Rollo (2018a) traces the development of this homology from ancient Greece and the Roman empire, through Augustinian Christianity, the Spanish colonisation of the Americas, and into Western social and political theory. For Rollo, the child forms the model through which colonisers aim to educate colonised peoples and facilitate their ‘progress’ towards civilisation.

Similarly, Nandy (1983, p. 11) argues that the aged homology between childhood and colonised peoples is a ‘subsidiary’ of the gendered homology between sexual and political dominance within colonialism. Nandy (1984, p. 363) contends that, with the transformations of the seventeenth century—including ‘the consolidation of colonialism’—a clear demarcation between adulthood and childhood emerged. According to the new theory of progress, childhood was reconstructed as an inferior age of development towards maturity and manhood. For Nandy (1983, p. 15), colonialism picked up this theory of social progress and established an homology between the life‐cycles of individuals and societies. Marx, for example, tied primitive communism (hunter‐gatherer societies) to childhood innocence, and constructed history as a movement from infantile communism to adult communism. Freud's early followers, in turn, studied ‘primitive’ societies to uncover the secrets of childhood (Nandy, 1983, p. 13). Through this paternalistic paradigm, British colonisers in India invoked dual strategies of developmental reform and punitive administration, with the aim of orienting Indian progress towards a homogenous global civilisation (Nandy, 1983). Such strategies were not unique to India; Nandy (1984, p. 360) highlights, for example, Cecil Rhodes' strategic depiction of Indigenous African peoples as dangerous children.

The paternalistic paradigm maps the field of possibilities through which the coloniality of age is ordered; it is a universal, linear, teleological and developmental timeline of growth upwards from the Black queer past of childhood towards a pre‐determined White cisheteronormative adult future. Whether one conceives of coloniality or the human/child distinction as primary, these analyses highlight the relationship between race and age (Nakata & Bray, 2023, p. 307). Against this background, colonisers construct the development of Black, Indigenous, and other colonised peoples as the problem, and pose the colonisers' paternalistic management of their growth upwards as the pathway to ‘racial justice’ and ‘decolonisation’. As a phenomenological experience of childhood's developmental time, growing up is produced through overlapping colonial chronopolitical formations, especially the patriarchal family (institutionalised in marriage) and educational institutions, such as kindergartens and schools (Halberstam, 2005; Kromidas, 2019b, 2022; Stewart, 2019). Building on this critique of developmental time, the coloniality of age problematises the colonial development of Black, Indigenous, and other colonised peoples by questioning its ‘upwards’ orientation towards ‘modern civilisation’. It locates ‘growing up’ within the chronopolitical formations of coloniality: imperialism, racial capitalism, settler colonialism, and so on. Within this framework, the orientation of this growth—upwards—marks a specific direction of movement out of a Black past towards a normative White future.

## 5 THE CHRONOPOLITICS OF BLACK CHILDHOOD

In the preceding sections, I have argued that the coloniality of age is organised through the doctrine of *tempus nullius* and the paternalistic paradigm. The coloniality of age naturalises Black childhood as a particularly intense site of colonial surveillance and violence (Breslow, 2019; Rollo, 2018b). In colonial constructions, Black childhood is figured as a liminal and queer age of adolescence. The doctrine of *tempus nullius* works to eliminate Black trajectories of growth; Black children's bodies are therefore represented as overdeveloped, strong, and hypersexual (Dancy, 2014). The infantilisation of the Black race through the paternalistic paradigm simultaneously works to limit Black children's growth upwards into adulthood; Black peoples' minds are therefore represented as childish, savage and capricious (Breslow, 2019). Black childhood is alienated not only from being, but from futurity. As Sharpe (2016, p. 79) argues, Black children live in the afterlife of the slave‐law of ‘partus sequitur ventrem (again, “that which is brought forth follows the womb”)’, which stated that children inherit the slave ‘non/status’ of their mother. This alienates Black childhood from Black temporal relations to their mother, family, inheritances, ancestors, and descendants (Teshome & Yang, 2018). Black children are narrated as a threat to White futurity, most commonly as violent criminals from dysfunctional and disconnected families (Shange, 2019). The coloniality of age renders the futurity of Black childhood unthinkable and impossible; Black childhood constitutes the temporal limit of coloniality.

In contrast to abstract deconstructions of time, the coloniality of age orients the analysis towards the temporal limits of coloniality. By locating racialised time within the colonial and patriarchal chronopolitical formations through which it emerges, the coloniality of age presents development or growth as a product of movement through multiple, complex, and dynamic social and political relations. The coloniality of age unsettles the temporal frame of coloniality itself. It acknowledges the temporal limits that colonial violence imposes on Black children's possibilities, but recentres their enfleshed temporal agency independently of the colonial matrix of power (Shange, 2019; Spillers, 1987). While Black children are racialised, gendered, and sexualised subjects, they may also inhabit otherwise worlds; chronopolitical formations and histories of family, community, and diaspora with their own histories and futures (English, 2018; Kromidas, 2019b, 2021). Such otherwise chronopolitical formations may facilitate Black children's temporal agency, providing them with the resources required to navigate the world according to their own timeframes and temporal goals (Conrad, 2020). As a theoretical framework, the coloniality of age opens up the possibility that Black children might turn away from White futures, and instead choose to grow otherwise. Growing otherwise signifies movement in directions other than ‘up’; movements towards otherwise futures beyond the temporal limits of coloniality. This article is only intended to gesture towards such possibilities.

This article therefore applies the coloniality of age to qualitative research into the presence of Black childhood as a race‐troubling figure in Britain. I undertook counter‐narrative interviews and analysis with young Black people in Britain. Counter‐narratives have long been used in anti‐racist research and activism to empower silenced peoples and trouble hegemonic narratives in a ‘war between stories’ (Bell, 1987; Delgado, 2013, p. 72; Wynter, 2003). In narrative, ‘a speaker connects events into a sequence that is consequential for later action and for the meanings that the speaker wants listeners to take away from the story’ (Reissman, 2008, p. 3). Narrative's focus on sequence allows for lived experiences of delay and growth over time that are central to unpacking the coloniality of age. Rather than trying to make Black children intelligible in ways that reify colonial structures, counter‐narratives restore the indeterminacy of Black children's temporal agency in navigating and forging multiple lines of flight, allowing possibilities for action to emerge from otherwise places. Each of the counter‐narratives form part of a broader story. This broader story speaks to the temporal coordinates of coloniality; it should not be taken as an abstract and universal trajectory of Black childhood.

I undertook semi‐structured counter‐narrative interviews with nine participants. Participants were recruited by contacting African and African Caribbean community groups and snowball sampling. The project was presented as an opportunity to challenge the Whiteness of supposedly ‘normal’ British childhoods, and to amplify the marginalised experiences of young Black people in Britain. Interviews took place between January and March, 2020. Interviews followed narratives important to participants around personal and family history, racialised identity, and belonging in Britain. Participants were required to identify as Black and of African or African Caribbean descent, to have lived in England for at least 5 years as a child, and to be between 18 and 25 years old. The counter‐narratives reflect the participants' reconstructions of their past in light of their present selves (Orellana & Phoenix, 2017). As ‘young adults’, their counter‐narratives problematise not only childhood but also ‘growing up’, the passage from childhood into adulthood. The most illustrative counter‐narratives have been included here, as produced in conversation with three interviewees. All names are pseudonyms and identifiable details are generalised to ensure participants' anonymity.

As a young White Australian settler of British descent, I co‐produce the counter‐narratives through the theoretical framework, interview, and analysis (Reissman, 2008, p. 22). From my White settler standpoint on sovereign Wurundjeri land, I see race as a product of the irreducible but inseparable histories of European imperialism and settler colonialism since at least 1492 (Kelley, 2017; King, 2019). The coloniality of age is therefore particularly informed by Black, Indigenous, and settler critiques of race. My interpretation of the narratives nevertheless acknowledges the specificity of Black African or African Caribbean peoples' experiences of childhood in Britain. My position as a White Australian researcher often led participants to provide thorough explanations of the British context and Black cultures; one participant seemed to deliberately refuse to partake in this dynamic. Participants also filtered their analyses and experiences of race and racism. Some were initially hesitant to name and discuss racism with me; some minimised the felt impact of racism. I likely missed some social cues (Gibson & Abrams, 2003). In consideration of my position in the interview, I worked to build rapport and trust, reiterated the anti‐racist purpose of the research, encouraged participants to talk freely, and asked for clarification where required. In the interpretation of the narratives, I pay close attention to euphemisms for race and racism, and other strategies that minimise racism's felt impacts. To centre the agency of the participants in my interpretation, I organised participants' experiences by forms of delay, growth, orientations, and open/closed futures, based in their own descriptions. Interviews were transcribed verbatim and analysed in this form to capture the original intent and meaning of the interviewee, with transcriptions edited here for clarity and brevity. Narratives of particular points in participants' lives collectively produced an overall life narrative. Connections between particular narratives and the life narrative were not always thematically explicit; structural analysis was therefore essential to understanding participants' experiences of delay and growth over time (Reissman, 2008).

## 6 ‘STUCK’

Many of the young Black counter‐narrators demonstrated awareness of the ways in which Black temporal agency is denied through the coloniality of age. The doctrine of *tempus nullius* works to limit Black childhood by robbing Black children of histories which they may take up, reconfigure, play with, and act on in the present. The Black past was imposed on Michelle as a child, producing a limit to her growth.

Michelle recalled being seen in a ‘very stereotypical way’ in primary school. She came to view herself through the racialised hatred of others; the negation of her Blackness was ‘interiorised or epidermalized, lived’ (Al‐Saji, 2013, p. 4).

Michelle When I was younger, I associated Blackness with things that were negative. In terms of how I viewed myself, when you see hatred towards your skin colour, I think subconsciously it made me hate that aspect of myself.

Michelle was predominantly raised by her White working‐class mother in southeast London; her parents separated when she was young, and she had a distant relationship with her Ivorian father and family. As a child, Michelle therefore had limited access to Black histories from within her family. Her epidermalization of racism was fuelled by the predominant depictions of Black peoples as lacking temporal agency at her school:

Michelle The only history you learn about Black people at school is slavery in America, and when it is mentioned, they just say ‘people did this to Black people’. There's no mention of resistance or anything like that.

Seeing people who look like you being oppressed, without showing the other aspects of it as well, really harms how you feel about yourself. It’s very damaging for a lot of people.

If you don’t have more knowledge through your parents or through a big standard institution like a pan‐African school, then you’re really stuck throughout the system.

Michelle was battered down by slavery in a similar manner to Fanon. In the histories presented to Michelle in school, Black peoples are without history; there is no mention of their temporal agency, even in resistance. Instead, Black peoples are presented as objects of empire. Michelle's education in the Black past therefore came to be a lived experience for Michelle; she was alienated from her own future because she lacked access to Black histories that she could take up as a resource for action. That Michelle is ‘stuck’ in this racialised education and social system reflects the temporal limit of coloniality institutionalised through the doctrine of *tempus nullius*; Michelle's school works to limit her growth. Michelle's references to gaining access to Black histories through parents or through other Black institutions like pan‐African schools, however, also indicates her awareness that resources for her temporal agency exist in other places. Michelle actively sought out resources outside school and university, such as Black histories of civil rights activism, that enabled her to embrace her Blackness.

## 7 ‘GROWING UP’

The coloniality of age works to organise Black children's experience of growing upwards as they follow the trajectory of the paternalistic paradigm. The denial of Black histories through the doctrine of *tempus nullius* produces the universal trajectory of growth as ‘upwards’ towards White futures. In response, Black children may adapt their growth in order to navigate the temporal limits of coloniality. A number of research participants, including Michelle, recalled making a strategic effort to grow up by minimising their racialised difference from White bodies. Through this strategy, participants sought to establish their belonging in Britain. Malik, for example, adopted this strategy upon arriving in Britain at the age of ten.

As a gay/queer boy of Muslim Somali background, Malik described his arrival in Britain as marking his ‘first transition towards growing up’. On his arrival, Malik attended a ‘White British’ school:

Malik Within school, playgrounds and stuff, you'd hear people using racial stereotypes about different groups, be that Asian, [or] Black. Specifically for me, being Somali, people would make comments about piracy scandals.

So in the beginning, school was a matter of trying to shape yourself into something ‘normal’. And I think that did genuinely lead to a denial of my Somali heritage.

Trying to be another model student also meant that it was a lot easier to form positive relationships with my teachers if I was like the rest of the students.

Callum What did you do to try and be ‘normal’?

Malik Things like speaking in a way that is seen as perfect English, shortening my name to make it easier for them to pronounce, and just adjusting myself in a way to mean that I was more acceptable to the people in my school.

The field of possibilities Malik faces in this narrative was already ‘anticipated, thought out, demonstrated, made the most of' (Fanon, 2008, p. 91; cited in Al‐Saji, 2013, p. 7), predetermined by the White body of the ‘model student’ upon his arrival in Britain. Malik's growth upwards is figured through his movement out of a Black past towards a White future, reflecting the paternalistic paradigm. In response to the limits imposed on Malik's practices through racist bullying, Malik made a conscious decision to alter his practices to appear more ‘normal’. This led, for Malik, to a denial of his Somali heritage. The imperative towards normality denies any imaginary variability to Malik, as seen in his account of language use. Where White British children are afforded the flexibility to engage playfully with language, Malik is aware that he must repeat the ‘perfect English’ of the model White student in order to leave behind his Black past and grow upwards. Malik's counter‐narrative denaturalises ‘growing up’, locating it within the colonial and patriarchal chronopolitical formations of the coloniality of age. In foregrounding his temporal agency in navigating his own growth within this context, Malik's counter‐narrative also acknowledges the possibility of another pathway for growth that he later took up: embracing queer Somali history.

## 8 THE ‘PACE’ OF RACISM

The coloniality of age produces a limit to Black children's ability to grow up to White futures. All of the research participants that recalled making a strategic effort to grow upwards also located this effort within broader counter‐narratives that showed an awareness of the limits of that pathway. As one research participant, Jerome, put it, ‘Ultimately, I was always Black, and that's what took over’. Many also acknowledged that some had more limited access to such a pathway, due to patriarchy and colourism. Adeyemi, a young woman of Nigerian descent, for example, situates her efforts to grow up in relation to the ‘pace’ of racism.

In her counter‐narrative of these limits, Adeyemi recalls her first workplace experience of an internship in a predominantly White office, an important moment of transition between childhood and adulthood:

Adeyemi When I interned in that place in the summer, there were no other Black people in the office, it was pretty much White. I felt very much from the get‐go, I wasn’t being put on projects. I think they just didn’t have any work for me.

In that situation, I would have really benefitted from having a Black person in the office, like, ‘How do I navigate this space? How do I speak up? How do I develop confidence? What are they looking for? What do they want?’

There were a couple of instances where I thought one particular person who was my senior was a bit shady. I didn’t trust her as much, but every time I would say ‘oh, I’ve run into this issue’, my colleagues would be like, ‘just speak to X person, she’s great’ and I was like, ‘ooh, I’m not getting the same…vibe’.

In a predominantly White workplace for the first time, and with no Black mentor to show her the way, Adeyemi struggled to develop confidence and learn how to navigate the racial dynamics of the White space as a young Black woman. From this experience, Adeyemi took away that:

Adeyemi No matter how hard you work to outperform in terms of college degrees and the skills that you can prove, it doesn’t really hold up in other aspects of your life. You still hit a ceiling, and aren’t promoted in the workplace.

I can have a very expensive, prestigious degree, and I can even land a very good job, but what happens once I get there? There is going to be stuff that I can’t‐ I can’t out‐ I can’t outpace racism. I can’t outpace all the kinds of disadvantages that I have. I just, I can’t.

And it’s exhausting to be like, ‘oh, I need to maybe dress in this way or talk in this way or laugh at these jokes’. I know lots of White people also try to ‘kiss up’ at work, and you also modify yourself. But I do think that as a Black person you just have to do so much more modification and I’m starting to see that in the long term, it might not be sustainable.

Adeyemi experiences the temporal limits of coloniality as a ‘ceiling’ preventing her promotion in the workplace. As Adeyemi recounts, she has worked hard to achieve many of the educational markers of growth upwards, including ‘a very expensive, prestigious degree’. However, upon reaching the adult workplace, Adeyemi finds that these educational markers are no longer enough. With a sense of exasperation that echoes Fanon's (2008, p. 91) ‘Too late!‘, Adeyemi realises that she ‘can't outpace racism’; due to the doctrine of *tempus nullius*, her White colleagues are always ahead of her. Adeyemi experiences growing up as a perpetual state of catching up that does not end with her transition into ‘adulthood’; her childhood is prolonged by her need to constantly modify herself to progress in the workplace. She tries to take up the predetermined possibilities for the future already determined by her White colleagues; she modifies her presentation in the ways she dresses, talks, or responds to jokes. Adeyemi acknowledges that White people also modify themselves, but not to the same degree. Here, Adeyemi is herself again modifying her presentation in an effort to outpace racism; she is speaking directly to me, the White man interviewer, and pre‐empting a negative response to her counter‐narrative. Through this counter‐narrative, Adeyemi contends that she is unable to keep pace with racism, no matter how much she modifies herself. As such, she is exhausted. Her acknowledgement that this repetition of White possibilities is unsustainable gestures towards a possibility of refusing the terms of White futurity, and instead growing otherwise.

## 9 ‘REGRESSING’

The coloniality of age limits what Black children may pick up and bring into the future. The paternalistic paradigm constructs history as a linear, progressive movement from Black pasts into White futures. As we have already seen, Malik's strategic efforts to grow upwards coincided with a denial of his Somali heritage. Many of the research participants also described their movement into White futures as requiring that they leave behind Black histories. Several of the research participants, however, described such a movement in terms of an alienating movement backwards. Michelle, for example, described her transition to a predominantly White university as ‘throwing me back to primary school’. Adeyemi similarly described her movement into a ‘very White’ university as a regression.

Adeyemi summarised this counter‐narrative as follows:

Adeyemi I guess what connects me to Nigeria is my skin, my skin tone. Especially going to a White primary school and then also coming‐ kind of going‐ regressing in the sense that I’m coming back to a very White institution [university] and you do that thing, especially as a woman, where you start to look at yourself and compare yourself to other women.

Adeyemi's struggle to find the right word to describe her history—‘coming‐ kind of going‐ regressing’—indicates the distinct nature of her growth. Her final description of her transition to a White university as a regression indicates a movement backwards to an earlier stage of development. Adeyemi unpacked this narrative of regression throughout her interview, which I here reorder chronologically.

Adeyemi I remember thinking to myself when I was younger, ‘you've got really weird features, you look like an alien’. It wasn't that I looked weird, it's just that, especially if you don't grow up in a community that looks like you, you think that the way you look is weird.

Adeyemi's insecurities are here tied to the racialisation of space. Because she attended a White primary school, Adeyemi was unable to recognise herself as belonging within her community. Her Black identity was negated, and she felt she looked ‘alien’. Adeyemi then went to a secondary school that she elsewhere describes as ‘quite racially diverse’:

Adeyemi I tried not to think about those things [beauty standards] too much when I was in secondary school…also education, again, because it was like ‘be smart, be smart’, so never thinking about those things.

In the racially diverse space of her secondary school, Adeyemi made an effort to set aside insecurities regarding beauty; she instead worked to identify her future positively through her intelligence. Adeyemi, however, then attended a predominantly White elite university:

Adeyemi I would say that coming to [a racially diverse school], then changing school ‐ and again, I had a similar thing to primary school where the [sixth form] school was Whiter and the teachers were predominantly White, and then coming to an elite university, the‐ I don't want to say that they were insecurities‐but the concerns I had about race in the UK very much manifested themselves.

Within the coloniality of age, Adeyemi's movement from primary school, to secondary school, to an elite university, appears as progress through normative stages of childhood development. Adeyemi's experience is, however, shaped by her confrontation with the temporal limits of coloniality. Adeyemi's movement in accordance with the paternalistic paradigm was tied to the negation of her Blackness. As such, in university, the ‘insecurities’ that Adeyemi experienced in the White space of her primary school reappeared; she once again compared her skin colour to her peers and felt ‘alien’. She therefore experiences her movement from a White space, into a racially diverse space, and back into a White space not as progress, but as a regression. In telling this counter‐narrative, however, Adeyemi takes up her history of belonging at a racially diverse school to displace her insecurities from her skin, and to relocate them in her ‘concerns about race in the UK’. This relocation reflects Adeyemi's temporal agency in reconstructing her non‐linear growth in terms of her future as a Black woman. Adeyemi locates her experience of feeling ‘alien’ at White institutions in the past, and locates her experience of belonging in racially diverse spaces as her future. In doing so, Adeyemi refuses the coloniality of age, opting instead to grow otherwise through her own temporal frame.

## 10 CONCLUSION

This article puts forward the coloniality of age as a theoretical framework that maps the colonial conditions of racialised time. It departs from descriptive genealogies of the coloniality of time, instead deploying age as an analytic to locate processes of becoming and possibilities for action within multiple, intersecting, dynamic, and complex networks of chronopolitical relations. The coloniality of age brings coloniality into the foreground as the predominant frame of reference for thinking ‘decolonisation’ and ‘racial justice’, while holding time open to possibilities that emerge from otherwise worlds. Michelle, Malik, and Adeyemi's counter‐narratives unsettle the coloniality of age. Their counter‐narratives restore Black children's temporal agency to the analysis as they navigate the chronopolitics of Black childhood. Their temporal agency broadens the horizon of possibilities beyond that predetermined by White bodies. The ‘impossible’ and utopian possibilities of otherwise worlds emerge and already exist in this age of Black self‐determination.

The coloniality of age is organised by the doctrine of *tempus nullius* and the paternalistic paradigm. The doctrine of *tempus nullius* produces a critical racialised distinction between the Black past and the White present that denies Black peoples' temporal agency. Without access to Black histories that she could make her own, Michelle found herself ‘stuck’ in the Black past. *Tempus nullius* locates White bodies ahead of Black bodies, leaving only a closed map of the future in their wake. This closed map of the future is produced through the paternalistic paradigm. The paternalistic paradigm models coloniality on the patriarchal adult/child relationship. The paternalistic paradigm directs the analysis to chronopolitical difference and temporal relations of growth upwards from a ‘primitive’ Black past to a ‘civilised’ White future. In his growth upwards towards a White future, Malik followed the developmental model of the White student and left behind his own queer Somali history. The coloniality of age offers a chronopolitical framework that complements geopolitical theories of *terra nullius* and the barbarian other.

The coloniality of age directs the analysis to the temporal limits of coloniality, constituted by Black childhood. The coloniality of age renders the futurity of Black childhood impossible. Colonial surveillance and violence works to deny the indeterminate futurity of Black children. Adeyemi's account of working hard to ‘outpace racism’ recalls a young Black woman coming up against the temporal limits of coloniality again and again, and consequently reconsidering her options. The coloniality of age, however, also opens the chronopolitics of Black childhood to possibilities of growing otherwise beyond the temporal limits of coloniality. In her second narrative, Adeyemi again comes up against the temporal limits of coloniality, this time experiencing her movement to a White university as a ‘regression’ to an earlier stage of her growth. Here, however, she reconfigures growth in terms of her growth as a Black woman, establishing her own distinct pathway to the future. Further analysis of the chronopolitics of Black childhood might follow this trajectory, or take ‘growing otherwise’ in new directions.

The coloniality of age aims to open up possibilities for otherwise worlds. Together, Michelle, Malik, and Adeyemi's counter‐narratives tell a story of Black children navigating colonial and patriarchal chronopolitical formations, confronting the temporal limits of coloniality, and refusing the coloniality of age in ways that generate other possibilities. Black childhood might then be reframed, not as an age with no future within the colonial matrix of power, but as an age with otherwise futures in the lifeworlds of Black, Indigenous, and other colonised peoples. From otherwise frames of reference, the construction of otherwise worlds might even appear both necessary and inevitable. If decolonisation and racial justice appear to be impossible and utopian demands, then perhaps we are looking for decolonial and just possibilities in the wrong age.

## CONFLICT OF INTEREST STATEMENT

No potential conflicts of interest with respect to the research, authorship, and/or publication of this article.

## ETHICS STATEMENT

Ethics approval granted by the University of Cambridge.

## PARTICIPANT CONSENT STATEMENT

All research participants provided their informed written consent to participate in this project.

## PERMISSION TO REPRODUCE MATERIAL FROM OTHER SOURCES

No permission to reproduce material from other sources was required for the research.

## Data Availability

Data supporting this study cannot be made available due to ethical restrictions.

## References

[bjos13141-bib-0001] Ahmed, S. (2006). Queer phenomenology: Orientations, objects, others. Duke University Press.

[bjos13141-bib-0002] Al‐Saji, A. (2013). Too late: Racialized time and the closure of the past. Insights, 6(5), 2–13.

[bjos13141-bib-0003] Beard, L. J. (2016). Decolonization and Indigenous sovereignty: Coming to terms with theories in the Americas. In J. G. Ramos & T. Daly (Eds.), Decolonial approaches to Latin American literatures and cultures (pp. 199–213). Palgrave Macmillan.

[bjos13141-bib-0004] Bell, D. (1987). And we are not saved: The elusive quest for racial justice. Basic Books.

[bjos13141-bib-0005] Breslow, J. (2019). Adolescent citizenship, or temporality and the negation of black childhood in two eras. American Quarterly, 71(2), 473–494. 10.1353/aq.2019.0039

[bjos13141-bib-0006] Burman, E. (2019). Fanon, education, action: Child as method. Routledge.

[bjos13141-bib-0007] Castro‐Gómez, S. (2021). Zero‐point hubris: Science, race, and enlightenment in eighteenth‐century Latin America. Translated by In G. Ciccariello‐Maher & D. T. Deere (Eds.). Rowman & Littlefield.

[bjos13141-bib-0008] Conrad, R. (2020). Time for childhoods: Young poets and questions of agency. University of Massachusetts Press.

[bjos13141-bib-0009] Crawley, A. (2020). Stayed | freedom | hallelujah. In T. L. King , J. Navarro , & A. Smith (Eds.), Otherwise worlds: Against settler colonialism and anti‐blackness. Duke University Press (Black Outdoors: Innovations in the Poetics of Study).

[bjos13141-bib-0010] Dancy, T. E. (2014). The adultification of Black boys. In K. Hasching‐Varner (Ed.), et al. (Eds.), Writing wrong. Sense Publishers.

[bjos13141-bib-0011] Delgado, R. (2013). Storytelling for oppositionists and others: A plea for narrative. In J. Stefancic & R. Delgado (Eds.), Critical race theory: The cutting edge (pp. 71–80). Temple University Press.

[bjos13141-bib-0012] English, D. K. (2013). Each hour redeem: Time and justice in African American literature. University of Minnesota Press.

[bjos13141-bib-0013] English, D. K. (2018). Race, writing, and time. In T. M. Allen (Ed.), Time and literature (1st ed., pp. 225–241). Cambridge University Press.

[bjos13141-bib-0014] Fanon, F. (2008). Black skin, white masks. Pluto Press.

[bjos13141-bib-0015] Gibson, P. , & Abrams, L. (2003). Racial difference in engaging, recruiting, and interviewing African American women in qualitative research. Qualitative Social Work, 2(4), 457–476. Available at:. 10.1177/1473325003024005

[bjos13141-bib-0016] Halberstam, J. (2005). In a queer time and place: Transgender bodies, subcultural lives. New York University Press.

[bjos13141-bib-0017] Kelley, R. D. G. (2017). The rest of us: Rethinking settler and native. American Quarterly, 69(2), 267–276. 10.1353/aq.2017.0020

[bjos13141-bib-0018] King, T. L. (2019). The black shoals: Offshore formations of Black and Native studies. Duke University Press.

[bjos13141-bib-0019] Kromidas, M. (2019a). “Agent of revolutionary thought”: Bambara and Black girlhood for a poetics of being and becoming human. Jeunesse: Young People, Texts, Cultures, 11(1), 18–37. 10.1353/jeu.2019.0001

[bjos13141-bib-0020] Kromidas, M. (2019b). Towards the human, after the child of Man: Seeing the child differently in teacher education. Curriculum Inquiry, 49(1), 65–89. 10.1080/03626784.2018.1549924

[bjos13141-bib-0021] Kromidas, M. (2021). Becoming convivial with child: Dismantling the race/child/ learning/human assemblage. In T. Kinard & G. S. Cannella (Eds.), Childhoods in more just worlds: An international handbook. Myers Education Press.

[bjos13141-bib-0022] Kromidas, M. (2022). Learning hierarchy and displacing conviviality: Time and subjectivity in the neoliberal kindergarten. Pedagogy, Culture & Society, 30(4), 491–510. 10.1080/14681366.2020.1817972

[bjos13141-bib-0023] Maldonado‐Torres, N. (2007). On the coloniality of being. Cultural Studies, 21(2–3), 240–270. 10.1080/09502380601162548

[bjos13141-bib-0024] Martineau, J. , & Ritskes, E. (2014). Fugitive indigeneity: Reclaiming the terrain of decolonial struggle through Indigenous art. Decolonization: Indigeneity, Education & Society, 3(1), I–XII.

[bjos13141-bib-0025] Mignolo, W. D. (2011). The darker side of western modernity: Global futures, decolonial options. Duke University Press.

[bjos13141-bib-0026] Mignolo, W. D. (2012). Coloniality at large: Time and the colonial difference. In S. Dube (Ed.), Enchantments of modernity: Empire, nation, globalisation (pp. 67–95). Routledge India.

[bjos13141-bib-0027] Mills, C. W. (2007). White ignorance. In S. Sullivan & N. Tuana (Eds.), Race and epistemologies of ignorance (pp. 11–38). State University of New York Press (SUNY series, philosophy and race).

[bjos13141-bib-0028] Mills, C. W. (2014). White time: The chronic injustice of ideal theory. Du Bois Review, 11(1), 27–42. 10.1017/s1742058x14000022

[bjos13141-bib-0029] Mills, C. W. (2020). The chronopolitics of racial time. Time & Society, 29(2), 297–317. 10.1177/0961463x20903650

[bjos13141-bib-0053] Muñoz, J. E . (2009). Cruising Utopia: The then and there of queer futurity. New York University Press.

[bjos13141-bib-0030] Nakata, S. (2015). Childhood citizenship, governance and policy: The politics of becoming adult. Routledge.

[bjos13141-bib-0031] Nakata, S. , & Bray, D. (2023). Political representation of Aboriginal and Torres Strait Islander youth in Australia. In B. Sandin (Ed.), et al. (Eds.), The politics of children’s rights and representation (pp. 301–323). Springer International Publishing.

[bjos13141-bib-0032] Nandy, A. (1983). The intimate enemy: Loss and recovery of self under colonialism. Oxford University Press.

[bjos13141-bib-0033] Nandy, A. (1984). Reconstructing childhood: A critique of the ideology of adulthood. Alternatives, 10(3), 359–375. 10.1177/030437548401000303

[bjos13141-bib-0034] Ngo, H. (2021). From “get over it” to “tear it down”: Racialized temporalities, “white time,” and temporal contestations. In L. Laubscher , D. Hook , & M. Desai (Eds.), Fanon, phenomenology, and psychology (pp. 194–208). Routledge.

[bjos13141-bib-0035] Orellana, M. F. , & Phoenix, A. (2017). Re‐Interpreting: Narratives of childhood language brokering over time. Childhood, 24(2), 183–196. 10.1177/0907568216671178 28503031 PMC5424852

[bjos13141-bib-0036] Pacini‐Ketchabaw, V. , & Kummen, K. (2016). Shifting temporal frames in children’s common worlds in the Anthropocene. Contemporary Issues in Early Childhood, 17(4), 431–441. 10.1177/1463949116677930

[bjos13141-bib-0037] Quijano, A. (1993). Modernity, identity, and utopia in Latin America. boundary 2, 20(3), 140–155. 10.2307/303346

[bjos13141-bib-0038] Quijano, A. (2000). Coloniality of power, eurocentrism, and Latin America. Nepantla: Views From South, 1(3), 533–580.

[bjos13141-bib-0039] Quijano, A. (2007). Coloniality and modernity/rationality. Cultural Studies, 21(2–3), 168–178. 10.1080/09502380601164353

[bjos13141-bib-0040] Reissman, C. K. (2008). Narrative methods for the human sciences. Sage.

[bjos13141-bib-0041] Rifkin, M. (2017). Beyond settler time: Temporal sovereignty and Indigenous self‐determination. Duke University Press.

[bjos13141-bib-0042] Rollo, T. (2018a). Feral children: Settler colonialism, progress, and the figure of the child. Settler Colonial Studies, 8(1), 60–79. 10.1080/2201473x.2016.1199826

[bjos13141-bib-0043] Rollo, T. (2018b). The colour of childhood: The role of the child/human binary in the production of anti‐black racism. Journal of Black Studies, 49(4), 307–329. 10.1177/0021934718760769

[bjos13141-bib-0044] Shange, S. (2019). Black girl ordinary: Flesh, carcerality, and the refusal of ethnography. Transforming Anthropology, 27(1), 3–21. 10.1111/traa.12143

[bjos13141-bib-0045] Sharpe, C. (2016). In the wake: On blackness and being. Duke University Press. Available at: http://ebookcentral.proquest.com/lib/unimelb/detail.action?docID=4717126. Accessed: 1 July 2024.

[bjos13141-bib-0046] Spillers, H. J. (1987). Mama’s baby, papa’s maybe: An American grammar book. Diacritics, 17(2), 65–81. 10.2307/464747

[bjos13141-bib-0047] Stewart, C. (2019). The future is queer kids: Queering the homonormative temporalities of same‐sex marriage. Politics, 40(3), 265–280. 10.1177/0263395719872595

[bjos13141-bib-0048] Sullivan, S. (2006). Revealing whiteness: The unconscious habits of racial privilege. Indiana University Press.

[bjos13141-bib-0049] Teshome, T. , & Yang, K. W. (2018). Not child but meager: Sexualization and negation of Black childhood. Small Axe, 22(3), 160–170. 10.1215/07990537-7249292

[bjos13141-bib-0050] Tuck, E. , & Yang, K. W. (2012). Decolonization is not a metaphor. Decolonization: Indigeneity, Education & Society, 1(1), 1–40.

[bjos13141-bib-0051] Wynter, S. (2003). Unsettling the coloniality of being/power/truth/freedom: Towards the human, after man, its overrepresentation—an argument. CR: The New Centennial Review, 3(3), 257–337. 10.1353/ncr.2004.0015

